# Supplementary material for: Interplay between bacterial deubiquitinase and ubiquitin E3 ligase regulates ubiquitin dynamics on Legionella phagosomes
Source: eLife. 2020 Nov 2;9:e58114. doi: 10.7554/eLife.58114 (PMC7669269; doi:10.7554/eLife.58114)
Supplement: Supplementary file 1. [file elife-58114-supp1.docx]

**Table S1** DALI search results against the PDB using the Lem27_1-417_ structure

| No. | PDB ID-Chain No. | Z scores | rmsd | %identity | Description |
| --- | --- | --- | --- | --- | --- |
| 1 | 4DDG-A | 10.4 | 2.32/143 | 13 | The human OTUB1/UbcH5b~Ub/Ub |
| 2 | 4BOU-A | 9.7 | 2.87/100 | 13 | Structure of OTUD3 OTU domain |
| 3 | 3PHU-B | 9.4 | 1.86/103 | 14 | OTU Domain of the Crimean Congo Hemorrhagic Fever Virus |
| 4 | 6KS5-B | 8.6 | 3.49/141 | 14 | The *L. pneumophila* deubiquitinase Ceg23 |
| 5 | 3TMP-E | 8.2 | 1.59/95 | 12 | The catalytic domain of human deubiquitinase DUBA/OTUD5 in complex with ubiquitin aldehyde |
| 6 | 5OE7-A  6SAK-A  6DRM-A | 8.2 | 2.63/128 | 11 | The OTU domain of Fam105A/OTULIN complex with Met1-linked diubiquitin |
| 7 | 3C0R-A | 7.7 | 3.38/75 | 12 | The OTUB1/Ovarian Tumor (OTU) domain in complex with Ubiquitin |
