# Supplementary material for: Interplay between bacterial deubiquitinase and ubiquitin E3 ligase regulates ubiquitin dynamics on Legionella phagosomes
Source: eLife. 2020 Nov 2;9:e58114. doi: 10.7554/eLife.58114 (PMC7669269; doi:10.7554/eLife.58114)
Supplement: Supplementary file 2. [file elife-58114-supp2.docx]

Table S2 Bacterial strains, plasmids and primers used in this study

| Bacterial Strains | Source | Identifier |
| --- | --- | --- |
| *L. pneumophila* (Philadelphia-1) LP02 | ([Berger and Isberg, 1993](#_ENREF_4)) | N/A |
| *L. pneumophila* LP03 | ([Berger and Isberg, 1993](#_ENREF_4)) | N/A |
| LP02Δ*lem27* | This study | N/A |
| LP02Δ*lem27* (pZL507) | This study | N/A |
| LP02Δ*lem27* (pLem27) | This study | N/A |
| LP02Δ*lem27* (pLem27_C24A_) | This study | N/A |
| LP02Δ*lem27*Δ*lotA* (pZL507) | This study | N/A |
| LP02Δ*sidCΔsdcA* | ([Hsu et al., 2014](#_ENREF_23)) | N/A |
| LP02Δ*sidCΔsdcA*(pZL507) | This study | N/A |
| LP02Δ*sidCΔsdcA*(pZL199) | This study | N/A |
| LP02Δ*sidCΔsdcA*(pZL199;pLem27) | This study | N/A |
| LP02Δ*sidC*Δ*sdcA*(pZL199;pLotA) | This study | N/A |
| *E.coli* BL21(DE3) | NEB | CAT#C2527I |
| *E.coli* XL1-Blue | Agilent | CAT#200249 |

| Plasmids | Source | Identifier |
| --- | --- | --- |
| pZL507 | ([Xu et al., 2010](#_ENREF_71)) | N/A |
| pJB908 | ([Sexton et al., 2004](#_ENREF_55)) | N/A |
| pZLQ | ([Luo and Farrand, 1999](#_ENREF_38)) | N/A |
| pZL507::*lem27* | This study | N/A |
| pZL507::*lem27_C24A_* | This study | N/A |
| pZLQ::*lem27* | This study | N/A |
| pZLQ::*lotA* | This study | N/A |
| p4xFlagCMV | ([Qiu et al., 2016](#_ENREF_48)) | N/A |
| p4xFlagCMV::*ub* | This study | N/A |
| p4xFlagCMV::*rab10* | This study | N/A |
| pSR47S | ([Luo and Isberg, 2004](#_ENREF_39)) | N/A |
| pZL199 (SidC on pJB908) | ([Hsu et al., 2014](#_ENREF_23)) |  |
| pSR47S::Δ*lem27* | This study | N/A |
| pSR47S::Δ*lotA* | This study | N/A |
| p4xFlagCMV::*rab10* | This study | N/A |
| p3xHACDNA3.1::*ub-11K* | This study | N/A |
| p3xHACDNA3.1::*ub-27K* | This study | N/A |
| p3xHACDNA3.1::*ub-29K* | This study | N/A |
| p3xHACDNA3.1::*ub-33K* | This study | N/A |
| p3xHACDNA3.1::*ub-48K* | This study | N/A |
| p3xHACDNA3.1::*ub-63K* | This study | N/A |
| pGFP::*sdeA_1-200_* | ([Qiu et al., 2016](#_ENREF_48)) | N/A |
| pGFP::*lem27* | This study | N/A |
| pGFP::*lem27_C24A_* | This study | N/A |
| pQE30 | Qiagen | CAT#32915 |
| pQE30::*lem27* | This study | N/A |
| pQE30::*lem27_C24A_* | This study | N/A |
| pQE30::*lem27_H304A_* | This study | N/A |
| pQE30::*sdeA* | ([Qiu et al., 2016](#_ENREF_48)) | N/A |
| pET28a | Novagen | CAT#69864 |
| pET28a::*ub* | ([Gan et al., 2019a](#_ENREF_15)) | N/A |
| pET28a:: *ubcH7* | This study | N/A |
| pET28a::*sidC_1-542_* | This study | N/A |
| pET28a::*lem27_(1-417)_* | This study | N/A |
| pET28a::*lem27_(1-417)D148A_* | This study | N/A |
| pET28a::*lem27_(1-417)Y149A_* | This study | N/A |
| pET28a::*lem27_(1-417)E151A_* | This study | N/A |
| pET28a::*lem27_(1-417)N210A_* | This study | N/A |
| pET28a::*lem27_(1-417)R213A_* | This study | N/A |
| pET28a::*lem27_(1-417)S243A_* | This study | N/A |
| pET28a::*lem27_(1-417)E244A_* | This study | N/A |
| pET28a::*lem27_(1-417)Q255A_* | This study | N/A |
| pET28a::*lem27_(1-417)E257A_* | This study | N/A |
| pET28a::*lem27_(1-417)R266A_* | This study | N/A |
| pET28a::*lem27_(1-417)I267A_* | This study | N/A |
| pET28a::*lem27_(1-417)E268A_* | This study | N/A |
| pET28a::*lem27_(1-417)I275A_* | This study | N/A |
| pET28a::*lem27_(1-417)N302A_* | This study | N/A |
| pET28a::*lem27_(1-417)Y149A S243A_* | This study | N/A |

| Primers | Sequence | Note |
| --- | --- | --- |
| pSL1001 | ctgggatccatggtgagggttttgaat | *lem27* 5F BamHI |
| pSL1002 | ctggtcgacttacattgcagcacgctt | *lem27* 3R SalI |
| pSL1003 | ctggtcgacttattcaaccataagggc | *lem27_(1-417)_* 3R SalI |
| pSL1004 | ctggtcgaccctttggcattatgggtg | *lem27*upSalI knockout |
| pSL1005 | ctgggatccattaaacatattgactcc | *lem27*upBamHI knockout |
| pSL1006 | ctgggatcccaagaatcaattagtgaa | *lem27*downBamHI knockout |
| pSL1007 | ctggagctcatcatcgaatattattgg | *lem27*downSacI knockout |
| pSL1008 | gaaatactgtaagcataatacaaggcattgcctttaccactattatccaca | *lem27 _C24A_* -1 |
| pSL1009 | tgtggataatagtggtaaaggcaatgccttgtattatgcttacagtatttc | *lem27_C24A_* -2 |
| pSL1010 | ggaatgatagaagtccaagcaacattgccttcattatttaatatcatttccgga | *lem27 _H304A_* -1 |
| pSL1011 | tccggaaatgatattaaataatgaaggcaatgttgcttggacttctatcattcc | *lem27 _H304A_* -2 |
| pSL1012 | ctgggatccatgatcagtttgggagaagcc | *sdeA* 5F BamHI |
| pSL1013 | ctggtcgacttaaaatcctatagtttt | *sdeA* 3R SalI |
| pSL1014 | cgagggtgatggtcttccccgtaagggtt | *ub-11K*-1 |
| pSL1015 | aacccttacggggaagaccatcaccctcg | *ub-11K*-2 |
| pSL1016 | tggatcctggcctttacattttctatcgtatccgag | *ub-27K*-1 |
| pSL1017 | ctcggatacgatagaaaatgtaaaggccaggatcca | *ub-27K*-2 |
| pSL1018 | cttccctatcctggatcttggcccttacattttct | *ub-29K*-1 |
| pSL1019 | agaaaatgtaagggccaagatccaggatagggaag | *ub-29K*-2 |
| pSL1020 | gaggaattccttccttatcctggatcctggcc | *ub-33K*-1 |
| pSL1021 | ggccaggatccaggataaggaaggaattcctc | *ub-33K*-2 |
| pSL1022 | agagactgatctttgctggcaagcagctggaaga | *ub-48K*-1 |
| pSL1023 | tcttccagctgcttgccagcaaagatcagtctct | *ub-48K*-2 |
| pSL1024 | tgaagagtagactccttttgaatattgtagtcagacaaagtacgt | *ub-63K*-1 |
| pSL1025 | acgtactttgtctgactacaatattcaaaaggagtctactcttca | *ub-63K*-2 |
| pSL1026 | ctgggatccatggcggccagcaggagg | *ubcH7* 5F BamHI |
| pSL1027 | ctggtcgacttagtccacaggtcgctt | *ubcH7* 3R SalI |
| pSL1028 | ctgggatccatggtgataaacatggtt | *sidC_1-542_* 5F BamHI |
| pSL1029 | ctggtcgacttaggtagcaaccaaataatc | *sidC_1-542_* 3R SalI |
| pSL1030 | ctgggatccatggcgaagaagacgtac | *rab10* 5F BamHI |
| pSL1031 | ctggtcgactcagcagcatttgctctt | *rab10* 3R SalI |
| pSL1032 | gtatatctcggcctcagtataagcaggattcgaaaaatcattgtc | *lem27_(1-417)D148A_* -1 |
| pSL1033 | gacaatgatttttcgaatcctgcttatactgaggccgagatatac | *lem27_(1-417)D148A_* -2 |
| pSL1034 | atatctcggcctcagtagcatcaggattcgaaaaatcattgtctattaaca | *lem27_(1-417)Y149A_* -1 |
| pSL1035 | tgttaatagacaatgatttttcgaatcctgatgctactgaggccgagatat | *lem27_(1-417)Y149A_* -2 |
| pSL1036 | ccttgtatatctcggccgcagtataatcaggattcga | *lem27_(1-417)E151A_* -1 |
| pSL1037 | tcgaatcctgattatactgcggccgagatatacaagg | *lem27_(1-417)E151A_* -2 |
| pSL1038 | tctatcgtttcttttcttaggatagcatctaaaatagtcgcctgatgaacc | *lem27_(1-417)N210A_* -1 |
| pSL1039 | ggttcatcaggcgactattttagatgctatcctaagaaaagaaacgataga | *lem27_(1-417)N210A_* -2 |
| pSL1040 | ctaataaaaaatctatcgtttcttttgctaggatattatctaaaatagtcgcctgat | *lem27_(1-417)R213A_* -1 |
| pSL1041 | atcaggcgactattttagataatatcctagcaaaagaaacgatagattttttattag | *lem27_(1-417)R213A_* -2 |
| pSL1042 | accatcagtgtttcttcagcaccccatacgaactctcttcg | *lem27_(1-417)S243A_* -1 |
| pSL1043 | cgaagagagttcgtatggggtgctgaagaaacactgatggt | *lem27_(1-417)S243A_* -2 |
| pSL1044 | taaaaccatcagtgtttctgcactaccccatacgaactc | *lem27_(1-417)E244A_* -1 |
| pSL1045 | gagttcgtatggggtagtgcagaaacactgatggtttta | *lem27_(1-417)E244A_* -2 |
| pSL1046 | ccatgcgttcacctgctatggcgcgatgtaaaaccatc | *lem27_(1-417)Q255A_* -1 |
| pSL1047 | gatggttttacatcgcgccatagcaggtgaacgcatgg | *lem27_(1-417)Q255A_* -2 |
| pSL1048 | gttacgtaccatgcgtgcaccttgtatggcgcg | *lem27_(1-417)E257A_* -1 |
| pSL1049 | cgcgccatacaaggtgcacgcatggtacgtaac | *lem27_(1-417)E257A_* -2 |
| pSL1050 | atcatagacaggctcaattgctccctcatggttacgtacc | *lem27_(1-417)R266A_* -1 |
| pSL1051 | ggtacgtaaccatgagggagcaattgagcctgtctatgat | *lem27_(1-417)R266A_* -2 |
| pSL1052 | atagacaggctcagctcttccctcatggttacgtaccatg | *lem27_(1-417)I267A_* -1 |
| pSL1053 | catggtacgtaaccatgagggaagagctgagcctgtctat | *lem27_(1-417)I267A_* -2 |
| pSL1054 | catgatcatagacaggcgcaattcttccctcatgg | *lem27_(1-417)E268A_* -1 |
| pSL1055 | ccatgagggaagaattgcgcctgtctatgatcatg | *lem27_(1-417)E268A_* -2 |
| pSL1056 | ctccatttctatgaacatgcaaaatagcttcatgatcatagacaggctcaattc | *lem27_(1-417)I275A_* -1 |
| pSL1057 | gaattgagcctgtctatgatcatgaagctattttgcatgttcatagaaatggag | *lem27_(1-417)I275A_* -2 |
| pSL1058 | caggaatgatagaagtccaatgaacagcgccttcattatttaatatcatttccg | *lem27_(1-417)N302A_* -1 |
| pSL1059 | cggaaatgatattaaataatgaaggcgctgttcattggacttctatcattcctg | *lem27_(1-417)N302A_* -2 |
| pSL1060 | ttaccttgtatatctcggcctcagtagcagcaggattcgaaaaatcattgtctatt | *lem27_(1-417)Y149A S243A_* -1 |
| pSL1061 | aatagacaatgatttttcgaatcctgctgctactgaggccgagatatacaaggtaa | *lem27_(1-417)Y149A S243A_* -2 |
| pSL1062 | ctgggatccatggcgaagacaattaag | *lotA* 5F BamHI |
| pSL1063 | ctggtcgacctaaggaacatatctgtt | *lotA* 3R SalI |
| pSL1064 | ctggtcgactgattatccctggataag | *lotA*upSalI knockout |
| pSL1065 | aattgcacttctaaaacagaaaacagacaagcgccatctcc | *lotA*upBamHI knockout |
| pSL1066 | ggagatggcgcttgtctgttttctgttttagaagtgcaatt | *lotA*downBamHI knockout |
| pSL1067 | ctggagctcaagcatctgcatgatacc | *lotA*downSacI knockout |
